# Time Course of Creativity in Dance

**DOI:** 10.3389/fpsyg.2020.518248

**Published:** 2020-12-15

**Authors:** David Kirsh, Catherine J. Stevens, Daniel W. Piepers

**Affiliations:** ^1^ Department of Cognitive Science, University of California San Diego, La Jolla, CA, United States; ^2^ MARCS Institute for Brain, Behaviour and Development and School of Psychology, Western Sydney University, Sydney, NSW, Australia

**Keywords:** creativity, improvisation, temporal dynamics of invention, iterative design, fail fast fail often, contemporary dance

## Abstract

Time-motion studies revolutionized the design and efficiency of repetitive work last century. Would *time-idea* studies revolutionize the rules of intellectual/creative work this century? Collaborating with seven professional dancers, we set out to discover if there were any significant temporal patterns to be found in a timeline coded to show when dancers come up with ideas and when they modify or reject them. On each of 3 days, the dancers were given a choreographic problem (or task) to help them generate a novel, high quality contemporary dance phrase. They were videoed as they worked on this task for sessions of 15, 30, and 45 min. At the end of each 15 min interval during each session, we had them perform the phrase they were creating. They recorded and then coded the video of themselves dancing during these sessions by using a coding language we developed with them to identify when ideas are introduced, modified, and rejected. We found that most ideas are created early and that though these early ideas are aggressively pruned early on, many still make it into the final product. The two competing accounts of creativity in design research make predictions for the temporal structure of creativity. Our results support neither account, rather showing a more blended version of the two. The iterative design view, arguably the dominant view, is that good ideas are the product of generating many ideas, choosing one fairly early, committing to it, and iteratively improving it. The “fail fast fail often” view is that good ideas are the product of rapidly generating and discarding ideas and holding back from early commitment to any one in particular. The result of holding back commitment, typically, is not that an idea is taken up later and then incrementally improved at the last minute, as much as that later designs are not completely novel, instead incorporating the best parts of the entire sequence of ideas. In our study, we found no evidence that one account or the other was more predictive for the domain of contemporary dance. The behavior of the dancers that we studied revealed elements of both, calling into question how predictive these theories are.

## Introduction

Is there a discernible temporal pattern in when good ideas arise in creative tasks such as dance choreography or designing a new chair? Do most ideas come early? Do the best ones come later?

In the literature on design there are two general views about creativity and its temporal structure. One view, associated with the philosophy of “Fail fast fail often,” holds that designers do best when they reject most ideas early on (Babineaux and Krumboltz, 2013). Failure, according to Ormerod (2005), is integral to human activity. The opportunity to learn and make something truly new is afforded by mistakes (Dennett, 2013). The assumption behind fail fast fail often is that by aiming for as many different designs as possible without a strong initial concern for which is the best, creators end up learning along the way and incorporating the best attributes of their diverse efforts into their eventual effort. Best comes later, it is thought, because later ideas inevitably build on what was good before.

This view is usually presented as a normative model. Good designers should keep the generation phase open as long as possible. Product design involves working in steps, typically, working through ideas on paper first then implementing them physically in some low fidelity pilot to get a sense of the realities of the idea. The approach of fail fast does not mean cutting out the initial pilot phase; it means that designers should not put their pilots to a test. It is enough to get a sense of the viability of a design by piloting it, but then without running actual tests to see just how good it actually is, designers are advised to return to the generation (paper) phase to invent new ideas. This recommendation applies even if the just-piloted idea has *prima facie* merit. The result from some studies is that the longer creative subjects work on ideating, the more interesting or complex their ideas become (e.g., Beaty and Silvia, 2012; Schwab et al., 2014). It is better to keep accumulating ideas and wait for feedback from tests to come all at once than accept test feedback on each idea immediately after it is piloted. In their work on engineering design, Dow et al. (2010) found that parallel feedback on all ideas is better than serial feedback one by one. Although “iteration can help people improve ideas. It can also give rise to fixation, continuously refining one option without considering others” (Dow et al., 2010).

The view that better ideas come later coheres with results found in purely experimental studies of creativity on formal creativity tasks, such as the remote associates task. First proposed by Guilford and his group (Christensen et al., 1957; Guilford, 1957), the serial order effect states that the more the mundane ideas tend to appear earlier, but get increasingly original, novel, and remote, the longer the subjects stay at the task. It is one of the oldest and most robust findings in modern creativity work (Beaty and Silvia, 2012). The finding is reminiscent of similar results in verbal fluency tasks (Thurstone and Thurstone, 1943; Rosen, 1980), where low-frequency words tend to appear later (e.g., Crowe, 1998).

Creative tasks in choreography and design, however, are importantly different from the divergent thinking tasks that Guilford (1957) studied. First, in ecologically natural design tasks, there is substantial interaction with media as subjects externalize their intermediate ideas during the process of working things out. In design tasks this working out is on paper or computer. In dance, it is *via* moving the body. Invariably, in real design tasks, there are surprising side effects between component parts of a design that are hard to anticipate without some externalization. Typically, a creative person will work on different aspects of a product (or a dance phrase) and then rely on seeing how they turn out. This takes time and involves tinkering. And the product is not a single word or idea but rather a collection of ideas that fit together. In classical divergent thinking experiments, by contrast, the tasks are formal, atomic, and brief – trials regularly take just 2 or 3 min. Often, there is no scratch pad, and on those occasions when writing is allowed, the target answer is a single word or phrase. Dancing is never an option in formal tasks. A second difference that calls the relevance of formal tests of creativity is that the test community – the subjects in Guilford style experiments – are not experts. The quick trials they participate in look quite unlike the activities that experts might spend many years of their lives mastering and improving. Those divergent thinking tasks are not the sort of thing that people devote their lives mastering.

The alternative view of creativity is that good design emerges from iteration. In iterative design, when a designer finds what seems to them to be a good paper idea – regardless of how early that idea might arise in the generation session – they should prototype it, and test it, then iteratively improve it on paper and prototype, test and prototype again. Design creation becomes essentially successive versioning. The evidence for iterative design vs. the competing fail fast view is to be found in how much time designers spend revising and improving an idea (in both paper and prototype) vs. how much time they spend creating new ideas in paper with a minimal prototyping and even less testing. Iterative design has the status of the received view.

The process of idea generation, more than the sequence of idea generation, has been investigated in contexts such as engineering (e.g., Daly et al., 2012; Starkey et al., 2016), business (Kline et al., 2013; Perry-Smith and Mannucci, 2015), and arts, including dance (Pressing, 1988; Stevens et al., 2003; Stevens and McKechnie, 2005; Kirsh et al., 2009; deLahunta et al., 2012; Dean and Bailes, 2016; Potter, 2018). In each case, it has been acknowledged that personal, social, and environmental conditions affect creative cognition, whether the domain be design, innovative thinking (e.g., Amabile, 1983; Paulus and Dzindolet, 2008; Boden, 2009), or art. This diversity of factors makes it challenging to settle on a generalizable set of determinants of creativity.

Studies more oriented to tracking idea generation in time, especially design oriented studies, typically resemble a head-to-head competition of the two positions, with each method being a strategy that subjects are assigned to follow (e.g., Dow et al., 2009, 2010). Typically, subjects are not experts. This leaves open the question of what expert subjects actually do when observed in naturalistic settings. Which strategy do they follow? It also raises the possibility that the null hypothesis is the best account. Namely, there is no pattern to be found in when good ideas are likely to arise. This may be because designers do not really follow an explicit strategy or because they change how they create on successive occasions. Possibly, individual differences between designers is so large that interpersonal regularities are not present to be found.

Our study involved recording and coding the creative process of seven company dancers from the Australian Dance Theatre (ADT) as they generated, selected, and rejected dance ideas. It was an ecologically natural process because they are experts, and their efforts were directed by the Artistic Director of the ADT Garry Stewart. Their task was to come up with potential dance phrases that Stewart might be able to adapt and later incorporate in the piece he was, at that time, making with the group. The creative method the dancers used in our study resembled their natural method in all but one respect: we manipulated the duration of their sessions to enable us to track the idea trajectory of each dancer and measure productivity during 15 min intervals.

Often, much of what a creative does is internal and is not visible from the outside. Fortunately, in dance most ideas are externalized, even nascent ones, since overt movement is the medium of both practice and performance (Humphrey, 1959; Hanna, 1979; Stevens et al., 2003). There is no paper phase and prototyping phase, so no separation between an idea on paper and transformation of that idea into more substantial material forms. The gap between imagining an idea and externalizing it is small. Even when dance ideas are ill-formed dancers explore properties of movement through externalizing and evaluating their interim products (Kirsh et al., 2009). Most scholars and dancers agree that manifesting ideas lies at the heart of creative work (Finke et al., 1992; Forsythe, 1999; Dean et al., 2006; deLahunta, 2007; May et al., 2011; Sawyer, 2012). So, external movement traces abound in dance, making it a good domain to study idea generation.

The challenge is that what is learned by a dancer by externalizing is rarely shared explicitly and much of it may be tacit. It is not always apparent to observers, even expert observers, when an idea germ first arises (Amabile, 1983; Runco, 1994; deLahunta et al., 2012; Sawyer, 2012). To overcome this problem, we relied on the dancers themselves to be the arbiters of new ideas. They served as our expert annotators; reviewing their own videos soon after each session and coding them using a coding language, we developed with them. We did not ask the dancers to code anything that went beyond what was presented in their videos, though clearly their recent memory of their movements helped them recognize idea generation and transformation. If they indicated an idea was rejected, however, it was because they visibly stopped working on it and did not return. External observers could recognize this same behavior once it was pointed out. If a dancer said they modified an idea, it was because it was clear that their performance changed in some externally verifiable way that added, subtracted, layered, or transformed the idea.

Even partial answers to the temporal order of ideation in dance, specifically, would be of substantial value in understanding how to design the work shifts of dancers. Many choreographers, for instance, have their dancers work in 60 or occasionally 90 min shifts (Kirsh et al., 2009). If more were known about when ideas emerge, it might be better to schedule breaks more often or have dancers review and consolidate their ideas at the end of 45 min, for example, rather than later. To date, little is known about the best way to manage time to optimize creativity in choreography nor is it better known in most other fields.

This lack of knowledge is due, in part, not only to the challenges of expert observation but also in part because the time course of idea generation has been less widely researched than the determinants and process of idea generation – the factors that are assumed to be the main cause of greater creativity. Examples include a study by Saggar et al. (2017) using fMRI to explore neural mechanisms in improvisation. They examined longitudinal changes in brain activity associated with participating in a 5-week design-thinking-based Creative Capacity Building Program (CCBP). Their conclusion was that improvisation is associated with reduced engagement of executive function and increased spontaneous implicit processing. As useful as this is, it leaves unexplored the question of the temporal order of in-session idea generation. Starkey et al. (2016) analyzed the way engineering students progress and filter ideas after initial idea generation. They observed that students tend to discard the most novel ideas they formed during concept selection in favor of conservative alternatives. Starkey and colleagues concluded that creativity at concept generation and selection does not predict creativity of the final design. However, their focus was on creative generation and filtering over an 8-week study, again leaving the question of what happens in a session unexplored.

Wang et al. (2017) used ERPs to study time course differences in perceiving creative vs. ordinary objects. Creative objects contain novel, creative information. They found that processing creative objects involves two stages: an early perceptual stage where visual differences of creative objects are detected and a later stage of understanding and encoding the creative information. This may be of interest for understanding audience perception of creative dance but leaves open the question of how long it takes to determine which of the two or more creative ideas is better.

In a series of articles, Magnusson (1996, 2000) has presented a method for identifying temporal patterns in data. Patterns and structures in movement and other sequences can be represented and quantified according to THEME (Magnusson, 1996, 2000). Events that make up a T-pattern can be described through a number of parameters including length, depth, or number of levels in an event tree, the times of occurrence of each component, distance between them, the number of actors involved, and how often the components alternated within each pattern (Casarrubea et al., 2015). T-pattern analysis enables detection of temporal structure, including recurring sequences, and is of particular relevance to self-organizing systems (Casarrubea et al., 2015, 2018).

T analysis using customized software has been applied in a range of domains and species. In the domain of dance, Torrents et al. (2011) applied it to dance improvisation under four conditions. Synchronization and interaction of dancers were detected and analyzed, revealing repeated sequences of events, the influence of interaction in pairs, and an association between breathing and group synchronization. In a study of contact improvisation, Torrents et al. (2010) detected T-patterns and event frequencies that revealed the influence of the partner on motor creativity and a concentration of motor skills on elevations, spatial changes, and turns.

T-pattern analysis can illuminate self-similar structure, cycling structure, hierarchical structure, and dyads as a mutually interacting system. In the present study, all movement is to be generated by dancers improvising alone, thus influences from interpersonal synchronization and interaction are not applicable. The emphasis is on the time course of events and the relatively coarse granularity of material (i.e., sans motion capture) may not lend itself to T analysis.

Time-motion studies changed industrial work, would *time-idea* studies change intellectual work?

Our goal in this study may be summarized like this: during their idea exploration sessions, are there statistically significant temporal patterns revealing a bias in when dancers come up with ideas and when they modify or reject them? Do they seem to follow a fail-fast strategy, an incrementalist strategy, or neither?

## Materials and Methods

### Participants

The sample comprised seven professional contemporary dance artists from the ADT (5M, 2F, mean age = 23.7 years, *SD* = 3.90; mean years of dance training = 13.6 years, *SD* = 5.5). The dancers formed the core company of ADT. This experiment was the fourth in a series of studies that took place over a 3-year period with ADT. Six of the seven dancers had participated in earlier unrelated and quite different experiments with the research team.

### Stimuli

The experiment consisted of five phases: (1) introduction to the experiment; (2) pilot task; (3) discussion about the pilot task and co-designed coding framework for annotating improvised material; (4) explanation of the three choreographic tasks, time periods, and allocation; and (5) debrief and discussion with the Artistic Director and researchers.

#### Introduction to the Experiment

Researchers and participants met as a group in the ADT studio together with the Associate Artistic Director and discussed the background to the experiment. DK and CS introduced themselves and provided some context for behavioral experiments in cognitive science. DK introduced the broad topic of the time course in design thinking and suggested a pilot run for dancers to get a feel for the experiment procedure and the dancers’ roles and tasks.

#### Pilot Task

DK described a broad focus on the time course of creating/improvising material that could subsequently be selected for a new work by ADT. Relevant questions, for example, include do the ideas come early and get developed, or do ideas come out in different time periods? He noted too that it may be the case that everyone is different; he emphasized that, in the experimental tasks, there is no right or wrong. It was explained that, in the pilot, dancers will work on a task individually and without looking at each other. Dancers were asked to call out “Now” when they think they have an idea germ or a new idea. Specifically, dancers were asked to “Generate and explore movement for a total of 15 min, recorded on an SD card. At the end of the 15 min, review the recording in QuickTime – identify what you found easy or hard. During our later conversation, we will try to sort out a vocabulary to see if we can develop a viable coding language.”

Associate Artistic Director Elizabeth Old gave instructions for the choreographic tasks that served as the pilot: “to make a phrase that expresses two contrasting states of being and all your images derive from biology and nature.”

#### Discussion About the Pilot Task and Co-Designed Coding Framework for Annotating Improvised Material

Dancers were asked if the task and protocol were feasible and they agreed that it was. The discussion included observations such as an idea not being recognized as such until “you have actually come up with something” that is “explored for a little while.” In the actual experiment, dancers rarely if ever called out “Now” for this reason – a new idea was not evident until some development had taken place and some potential recognized.

DK raised the coding and language. Terms were discussed that had been suggested by the dancers including “candidate, snowball, lightbulb moment, dynamics.” Dancers agreed that the coding is “doable.” For example, they commented on ideas when they “switched,” and appraised an idea as “good,” or “not good.” Dancers noted that movement tended to be more interesting when they were not saying “Now”; they wrote down every shift in a dynamic or idea. The importance of including the time stamp during the dancers’ coding or annotation of material was mentioned by the research team. One dancer commented that they remembered the idea as they reviewed their recording. In short, the task and coding were deemed doable.

**Figure 1:**
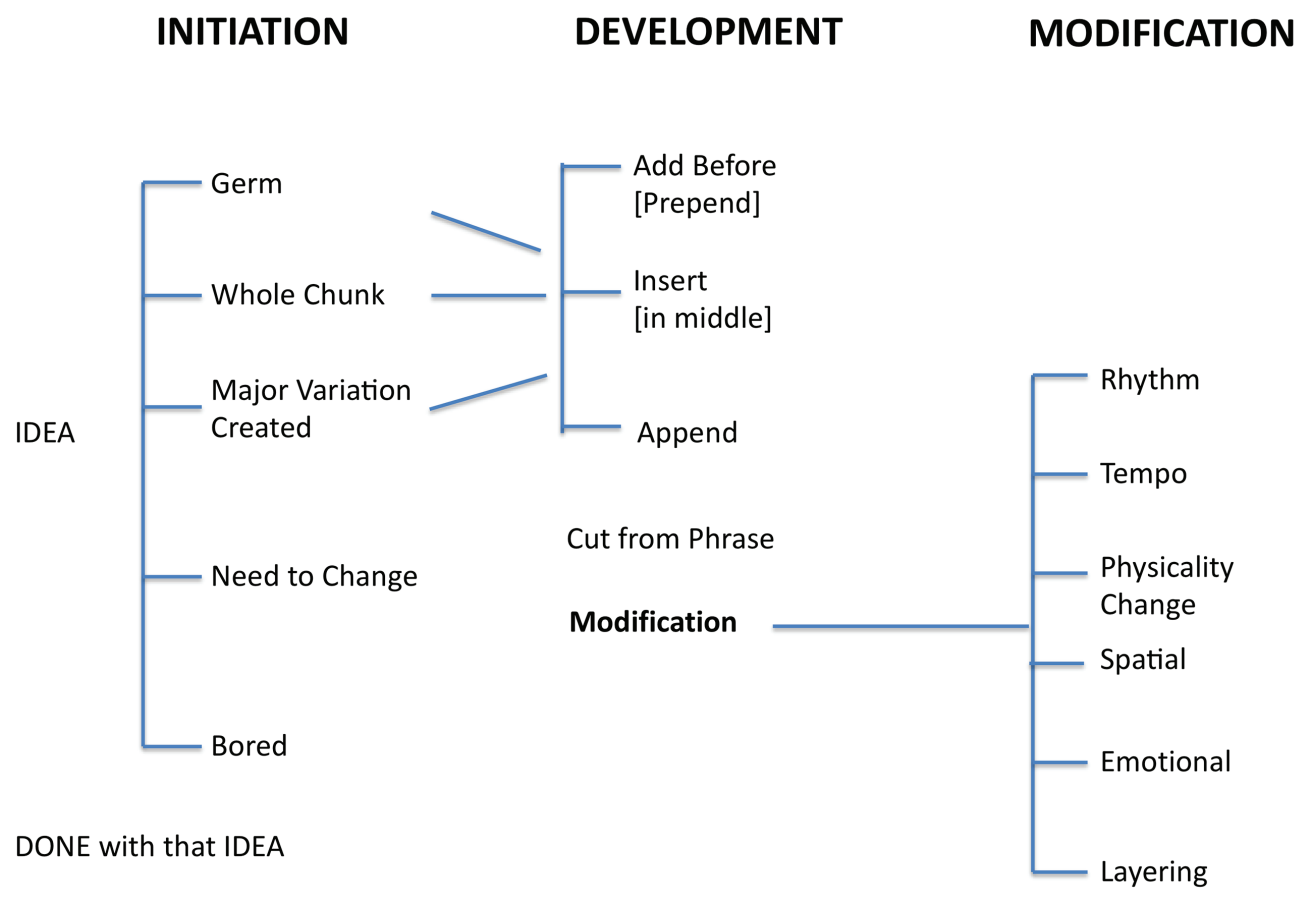
Coding scheme dancers used to self-annotate their videos. The focus was on idea generation and idea evolution during their improvisation tasks.

Terms were then collated from the discussion for an agreed coding framework. Dancers suggested terms especially in changing movements – “tempo, space, shapes, direction, shift of levels, rhythm, inversion, lightbulb, layering, change, bored, modification, variation, and add on.” The terms were sketched by DK together with the addition of some structures such as nesting and hierarchy, which the dancers also affirmed and to which the dancers contributed. The coding framework that emerged from the post-pilot discussion with the dancers is shown in Figure 1.

#### Explanation of the Three Choreographic Tasks, Time Periods, and Allocation

Once the pilot task had been conducted and discussed, the actual experiment began. There were three trials, each involving a different improvisation task. As before, the tasks were developed by the company’s Artistic Director and associated with a new work called “Nature.” Each task offered an inspirational idea and, as the Associate Artistic Director explained, the job of the dancers was to explore that idea through movement. The ideas/tasks were: (i) The body is a clock: it has circadian or longer rhythms, i.e., sleep-wake, day-night, tides, seasonal shifts; the body is a rhythmic system, so it needs rest to continue so it cycles between rest and activity; (ii) to represent senescence: the Second Law of Thermodynamics, tendency to randomness, losing the capacity to hold structure, hold information, and to carry information: life, death, decay; and (iii) to take a movement phrase already worked on, and alter layer, or vary it by incorporating an idea of moving through water as if one is water bound, or traveling on land – crawling, hopping, walking etc., or transform it by incorporating states like being frozen, being ancient, near death, or sightless. With Task 3, the Associate Artistic Director emphasized that the Artistic Director’s particular interest was in the shift or transition from one state to another.

#### Debrief and Discussion With the Artistic Director and Researchers

After the experiment, the dancers were invited as a group to answer the following questions:

1. Did you learn something useful or interesting about your practice?
2. Was there anything surprising?
3. Did you learn anything about how long you should work on a task before taking a break and coming back at another time?
4. Did it make sense to try to interpret your different shows as made up of phrases? Was it revealing?
5. Did you learn something interesting about tasks that work best for you? For instance, something about their generalness or the time needed to get something of quality out of them. Do you think you can predict in advance which tasks will be most productive for you?
6. Concerning Task 3 – working on a phrase you already developed:

- Do you think it was simpler than the others?
- Was it as generative or fertile, leading you to as many creative results as the other tasks?
- Were your products majorly different from the original pre-made you started with?

### Equipment

Each dancer’s creative process was recorded on an SD card in a JVC Everio full HD camcorder mounted on a Manfrotto tripod. Contents of the SD card were viewed on a laptop as dancers wrote in a notebook their timeline and a description of the movement material.

### Procedure

Participants received an information sheet and provided written consent (Western Sydney University Human Research Ethics Committee Approval No. H10527). The experiment was part of a collaborative project with ADT taking place in the ADT studios in Adelaide, Australia over 3 consecutive days. The dancers and Associate Artistic Director were told about the background to the experiment. DK then described the aims of the experiment and the need for a pilot task to investigate the feasibility of the experiment and clarity of instructions and procedure. During the pilot and actual experiment, the dancers worked individually without music in an area in one of three studio spaces. Because the dancers were familiar with the venue and their own dancing habits, they set up their cameras, positioning them in ways they knew would work best given how they typically stage their performances. Their objective was to video record their process of movement creation and movement exploration.

Dancers were asked to say “Now” when they explicitly began working on a new movement idea. After the 15-min pilot task, the dancers, researchers, and Associate Artistic Director discussed whether the procedure worked. The dancers then individually reviewed their own video recording of their 15 min of improvisation and produced a timeline of idea generation and development. The group reconvened to discuss the movement/idea coding process and brainstorm articulation of a shared and agreed coding vocabulary to use when annotating their recordings from the actual experiment trials. The immediate self-review of video footage minimized the role of memory and eliminated interpretation, misinterpretation, or value judgments by another person of material “quality” or creativity. The agreed nomenclature was developed collaboratively to maximize validity. As a group, everyone agreed to the meaning of coding terms and how to apply them in a consistent way.

The experiment was designed to measure idea generation, evolution, and abandonment over three time intervals (15, 30, and 45 min). A Latin Square design was used that crossed the three improvisation tasks (A–C) with the three time intervals, thereby enabling the use of repeated measures on each variable (First Run in Table 1). Participants were advised that they would complete each of the three tasks for each of 15, 30, and 45 min (i.e., for a total of nine trials) and that they would need to video record and code each trial. Task 1 was presented on Day 1 with dancers distributed over the three time intervals. After the trial each dancer immediately sat down to code their recording using the agreed upon coding language. On their own laptop, they applied the code to a timeline showing when they generated, changed, developed, or abandoned ideas or germs of ideas. Tasks 2 and 3 in various time intervals were presented on Day 2. Owing to time pressure, the time interval conditions that were not completed on Days 1 and 2 were collapsed on Day 3 by having all of the dancers work for 45 min straight on their task but stopping at 15, 30, and 45 min to “show” the material that they had developed up to that time; see second Run in Table 1. Each trial on Day 3 commenced with a “show” of material that had been created in the tasks on Days 1 and 2.

**Table 1:** Study design showing time period, task, dancer, and first and second (compressed) run of trial types.

	Time per condition					
	First run			Second run (15 min intervals)		
Dancer #	Task A	Task B	Task C	Task A	Task B	Task C
1	30	45	15	30	15	45
2	15	30	45	45	30	15
3	45	15	30	15	45	30
4	45	15	30	15	45	30
5	30	45	15	30	15	45
6	15	30	45	45	30	15
7	15	30	45	45	30	15

After data collection and video annotation was complete, the dancers and Artistic Director discussed the six questions that the researchers had posed about the experience of completing the experiment.

## Results

### Video Analysis

To acquire usable data, we had the dancers work under camera and perform a visible “core dump” – a performance of their interim ideas after 15, 30, and 45 min. The data consist of a time sequence of descriptions produced by the dancers themselves as they reviewed their own videos and annotated them by identifying the moments when they believed they introduced, modified, or rejected ideas. Other attributes were also coded for, but given the sparseness of the data, our objective was to determine if the data lent support to one of the two theories: iterative design vs. fail fast fail often.

Previous work on temporal patterns used THEME and T-pattern analysis to detect structure and recurring sequences (e.g., Magnusson, 2000; Castañer et al., 2009; Casarrubea et al., 2015). In the absence of motion capture or fine-grained movement coding, T-pattern analysis was not applicable to the present dataset.

Data were obtained from dancers’ annotated timelines for each trial and checked by the experimental team against the video recordings. The “show” videos were also reviewed and compared for the estimation of ideas and change. Three dependent measures were computed: the number of ideas generated in each of the three time intervals, the number of ideas retained from each of the three time intervals, and the number of ideas rejected in each of the three time intervals. Table 2 presents an overview of the total number of ideas generated, retained, and rejected in each of the three time intervals.

**Table 2:** Total number of ideas generated, rejected, and retained during 0–15, 15–30, and 30–45 min time intervals (percentages in parentheses).

Time intervals				
No. of ideas	0–15 min	15–30 min	30–45 min	Total
Created	83 (76.15)	18 (16.51)	8 (7.34)	109
Retained	53 (70.67)	16 (21.33)	6 (8.00)	75
Rejected	29 (85.29)	1 (2.94)	4 (11.76)	34

#### Do Most Ideas Come Early?

**Figure 2:**
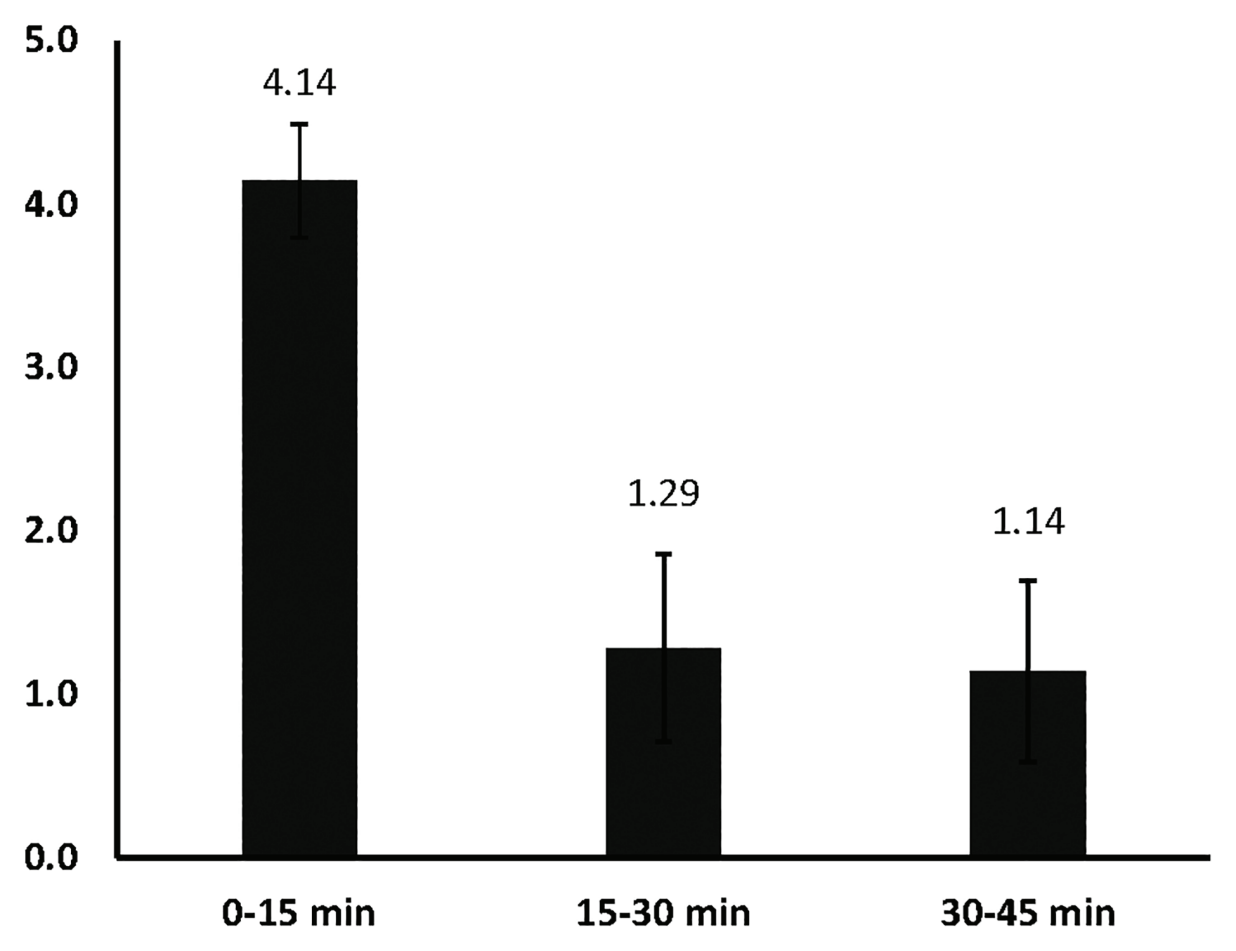
Mean number of ideas generated for each time interval.

Figure 2-the mean number of ideas generated in each of the time intervals indicates that most ideas do indeed come early *F*(2,12) = 14.58, *p* = 0.001, *η*_p_^2^ = 0.71 There was a significantly greater mean number of ideas generated in the 0–15 min interval (*M* = 4.14, *SD* = 0.92) than in both the 15–30 min interval (*M* = 1.29, *SD* = 1.52) and the 30–45 min interval (*M* = 1.14, *SD* = 1.46), *t*(6) = 5.03, *p* = 0.002 and *t*(6) = 4.19, *p* = 0.006, respectively. In fact, dancers generated as many new ideas in the first interval as they did in the next two combined. This suggests that more of their time in later intervals is spent iterating on earlier ideas or searching with less success for new ones. Put differently, the dancers come up with most of their ideas early and work with these over time.

#### The Number of Ideas Retained

**Figure 3:**
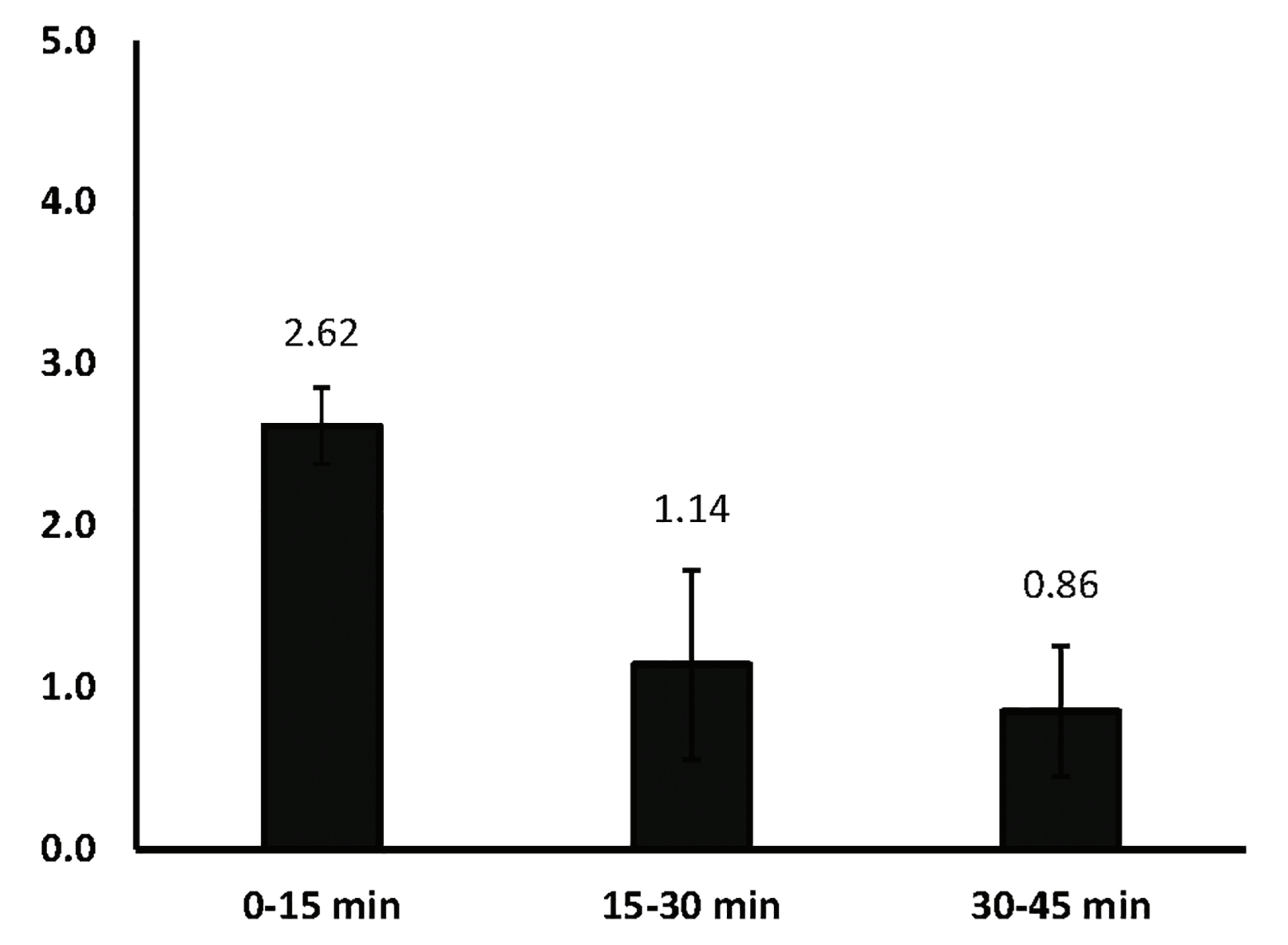
Mean number of retained ideas from each time interval; this refers to the number of ideas generated in the interval and not rejected.

How many of the ideas that are created in an interval remain alive by the end of that interval? That is, how many ideas are created in that interval and not rejected? Figure 3 shows how many ideas are retained and added to the sum of potentially viable ideas – ideas that might make it into the performance at the end of 45 min. The mean number of retained ideas in the 0–15 min interval was (*M* = 2.62, *SD* = 0.62); in the 15–30 min was (*M* = 1.14, *SD* = 1.55); and in the 30–45 min interval was (*M* = 0.86, *SD* = 1.07), *F*(2,12) = 6.81, *p* = 0.01, *η*_p_^2^ = 0.53, (Figure 3). Statistically, the mean number of ideas retained in the first interval is significantly greater than the numbers kept in the 15–30 min interval *t*(6) = 4.05, *p* = 0.007 and in the 30–45 min interval *t*(6) = 3.89, *p* = 0.008. This suggests that fewer viable ideas are being created as time goes on. But, we cannot tell whether dancers are making sub-threshold modifications to their live ideas, so they are iteratively improving them, but in small ways, or they are searching with diminishing success for new ideas.

#### How Soon After Inventing an Idea Do Dancers Reject It?

**Figure 4:**
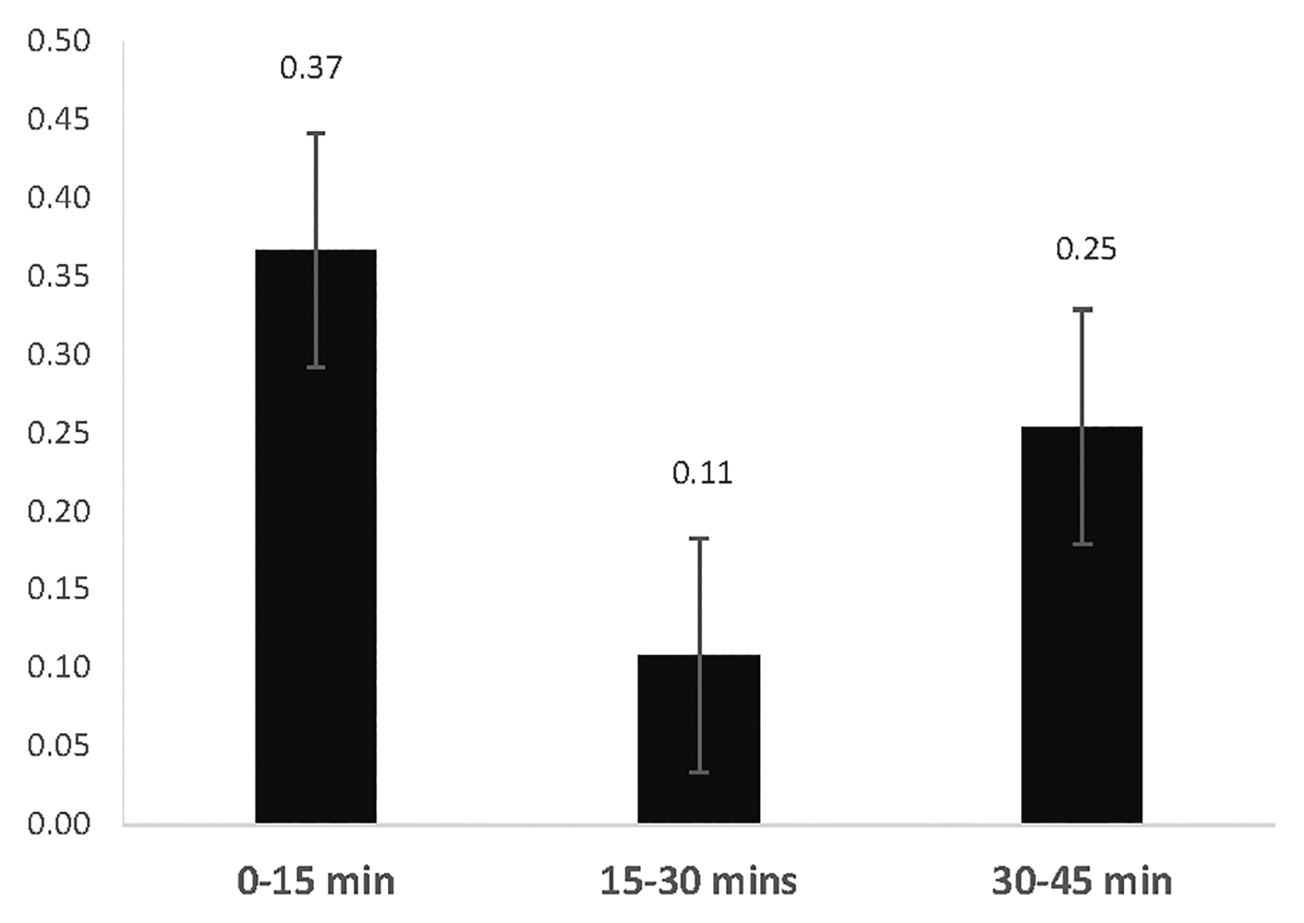
Mean number of rejected ideas from each time interval.

**Figure 5:**
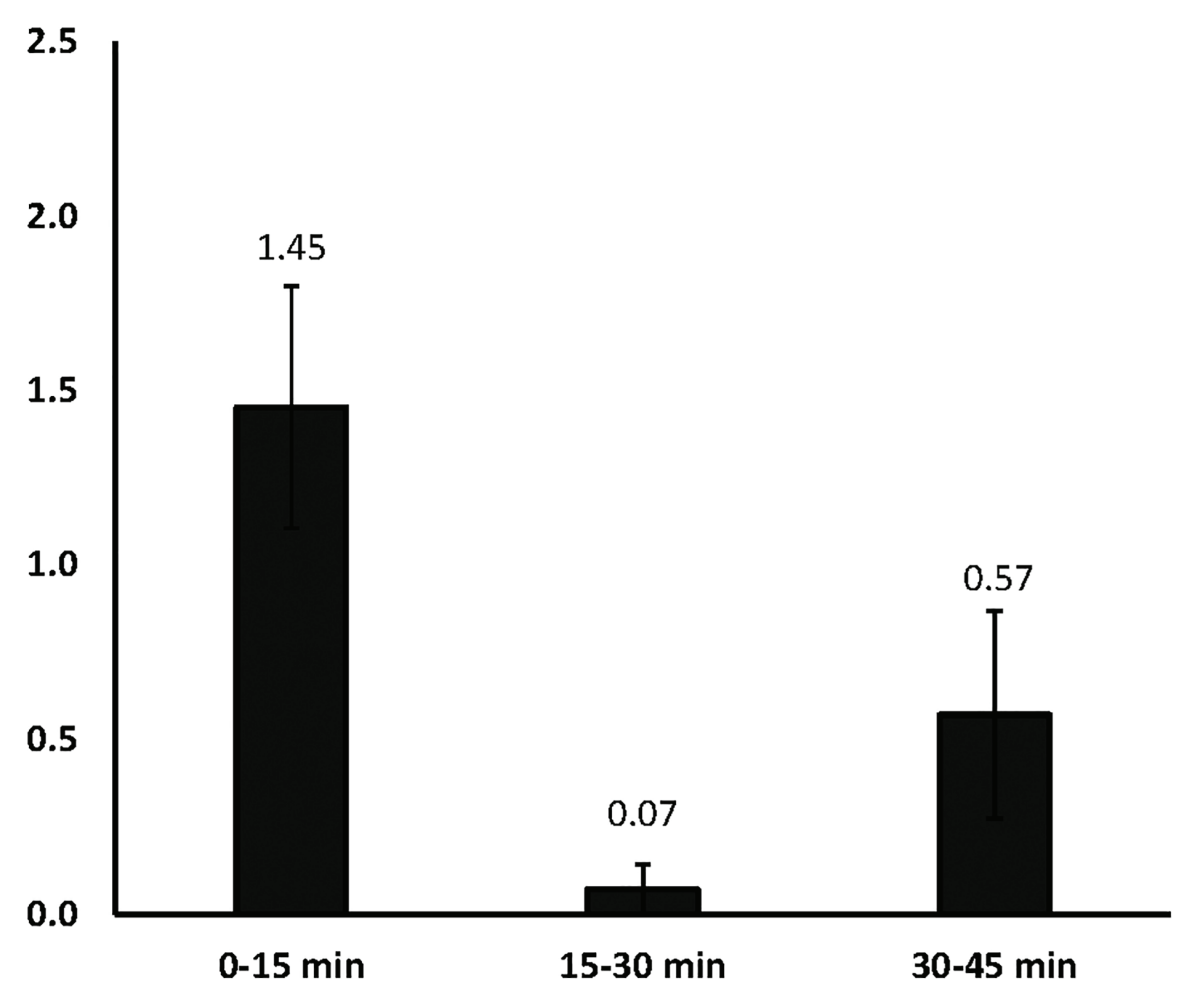
Percentage of ideas generated in an interval and then rejected in that same interval.

Figure 4 shows the mean number of rejected ideas during each time interval and the rejection rate interpreted as the percentage of the total number of live ideas remaining at the end of that period. Given that most ideas are generated early, it is no surprise that most ideas – in absolute terms – are also rejected early, *F*(2,12) = 6.51, *p* = 0.01, *η*_p_^2^ = 0.52. The median rate of idea rejection was only 62 s and the vast majority (94.11%) of rejected ideas were abandoned during the same time interval in which they were created. The mean number of rejections during the 0–15 min interval (*M* = 1.45, *SD* = 0.92) was significantly larger than during the 15–30 min interval (*M* = 0.07, *SD* = 0.19), *t*(6) = 3.75, *p* = 0.010 but not the 30–45 min interval (Figure 5). These findings suggest that participants aggressively throw away ideas in the first interval and then throw away fewer ideas during the second interval, suggesting that their rate of rejection slows down. A slight increase in the number of rejections during the last interval likely reflects the need to make final decisions concerning what to include in the final performance.

### Post Experiment Focus Group With Artistic Director and Dancers

#### Summary

The themes that emerged from a discussion with the dancers align with the quantitative results. In summary, the dancers commented that the ideas came early and that they then gave time and resource to develop and play with the early ideas. The different tasks had an influence, with some dancers finding Tasks 1 and 2 more straightforward for creative processes. Task 3 with its emphasis on working with an existing, pre-made phrase, felt less creative, with more of an emphasis on recall and the use of devices for change such as layering. While ideas did come early, the time constraint of 15, 30, and 45 min was too short for the development and setting of material. A partner or other dancers would also be needed for the development phase. More detailed examples of comments and themes from the focus group will now be described.

All of the dancers identified things that they learned from the experiment: “I did not realize how much I improvised to make material” (D8). Ideas coming early were evident: “I’ll go with my first idea and then I’ll improvise around that idea to try and find the quality that I feel matches that idea the best…once I have kind of a set quality then I’ll … start setting it” (D4). The idea was “natural” commented one of the dancers, and to develop it through improvisation they then ask, “how can I distort it somehow or make it odd?” (D8). “You kind of get product from improvising. And then you play around with it…I also realize I kind of mark things through” (D4).

“I would generate basically certain ideas in the first chunk of time and then try and extend on them … So, it was in the first 15 min that I was more stimulated, and I generated more ideas” (D6); “I feel the same” (D4). Compared with the material that was “shown” in later sessions, one of the dancers commented that the improvisation “better represented what I was trying to say and then when I tried to set it, it was in the same realm but wasn’t as…natural” (D3) or “textured” (D1).

The quantity of material generated and “shown” was surprising to the dancers: “These are long, there’s a lot of material … each phrase was nearly 3 min each” (D8). Surprising too, was the influence of the external environment on the process including the camera, multiple things happening, and “the split studio” (D2).

The dancers observed two constraints. First, working alone: “I do not have that person to feed off…I could go for much longer with a partner or more people” (D8); “initially I had this idea and I thought ‘oh this could work best in a group’…I could not go a second more on that without other people working on it…I think solo and group is very different…” (D6). Improvisation without a “reference point,” such as a mirror or another dancer, made “re-finding” or “replicating” the improvisation difficult” (D5). This was a reason that “when I was adding on to a phrase I’d always go back to the beginning and try and see what would come naturally out of it” (D3). Two dancers discussed the way that phrases are developed by teaching them to another dancer, seeing a new version of it performed by that dancer and then manipulating that new version (D8, D6). The second constraint that the dancers noted concerned the unchanging space and location: “knowing you only could stay in a certain amount of space…you might just want to take a walk just to find somewhere new for a bit” (D5).

Although tasks will differ, “my main objective is to portray that task in the best possible way I can … [to] physicalize the integrity of that task the best” (D4). Individual differences or preferences were implied: “different tasks clicked differently with everyone” (D3). Dancers differed too in preferring to stop to code/annotate or to keep on going (D8). Three dancers agreed that they were not creating multiple phrases but one phrase and that it might last 3 min.

Imagery was raised as key in some tasks, hence its inclusion in coding: “I think we did play a lot with imagery” (D8). One dancer noted that tasks involving states engaged “a certain kind of texture…and it’s a layering device instead of a creative; …with states…there was nothing other than imagery that you could really go with” (D1). Another dancer commented that they liked “the idea of generating from a state and thinking of the state as more of a structured improv[isation]” (D7).

Working with something pre-made was regarded by the dancers as simpler: “I found that it was much easier [given] the timeframe that we had” (D2). The dancers agreed too that working with the pre-made was “less interesting” (D8), “it was not original” (D6), “a more indulgent way of doing a phrase” (D3), and a “start from halfway” (D4). They agreed that working only with a pre-made phrase was difficult to sustain in the 45-min period: “Yeah 45 on that one would be hard” (D4). In the Artistic Director’s words, “it does not matter how you make something so long as it works…there’s no rules…it’s just different ways of working at different times” (C1).

In discussing the differences between improvising and making material from a pre-planned basis, “Time was a huge factor…you have only got 15 min to come up with something!” (D3). The dancers and the Artistic Director theorized that shorter time periods worked for stimulating ideas but “to develop that idea you need longer” (C1). The product that arises from developing a pre-made phrase was regarded as different: “we might be familiar with a few parts and a few shapes … but by layering those states completely, [it] changes the rhythm and movement and quality” (D5).

Emotional states, unlike layering, were not so easy to put on top of pre-existing material “because the material is material” (D8); “it’s much richer than when it’s a phrase from before because we will always associate it to the original phrase” (D6); and memory or recall can intrude for a dancer “thinking about what’s next rather than just letting it take me” (D7).

## Discussion

The aim of the experiment was to discover regularities in the emergence, development, rejection, and elaboration of ideas. Our findings can be summarized like this: dancers are most creative in their first interval. They are most aggressive in pruning their ideas too, during that first interval. Nonetheless, many of those first ideas make it into the final performance. Hence, good ideas do come early. Later, ideas also make it to the final product, but there are fewer of these and the pruning rate is lower, especially in the second interval. Pruning rate increases in the final interval, likely because final decisions must be made about what to include in the final performance.

### Does This Tell Us Anything About Views of Creativity in Design?

Two views about creativity currently making the rounds in discussions of design thinking are that new products are the result of: (1) iterative design or (2) the outcome of generating many different design ideas and rejecting them quickly – fail fast fail often (Kline et al., 2013; Vedin, 2014). Both reflect an almost unavoidable penchant to see design as a quasi-evolutionary process where large numbers of candidates are selectively pruned, with parts from failed designs often becoming parts in new designs after modification.

The main difference between the iterative and fail often views concerns the timing of when a designer makes a commitment. In iterative design, a commitment to the base or primal design form – the idea to improve – is taken early. Then, the long road of hill climbing begins, as parts and relations are upgraded by repeated testing and evaluation. The final form, in principle, may be arbitrarily distant from the starting form, but the path getting there has been incremental, changing a feature here and there as evidence of imperfection accumulates and leads the creative agent to modify or redesign that part.

Designers who follow the fail fast view, by contrast, need not commit to a base form until they stumble on a candidate probably superior to what has come before. This can lead to late commitment. The rhetoric behind this view is that it should be easy to give up ideas and move on. Good designers are ones who keep coming up with new ideas, even if these are not big ideas individually or even if their previous ideas have not actually failed. The principle is to “throw things out there” to see if they float. This creative style is supposed to have a better risk-reward profile because it is thought to lead to more novel ideas. Thus, Babineaux and Krumboltz (2013), Kline et al. (2013), and Vedin (2014) all repeat the claim that the “fail fast and often” crowd “try new things, make mistakes and in doing so benefit from unexpected experiences and opportunities” (Babineaux and Krumboltz, 2013, p. 7). In practice, the emphasis, however, is more on generating new candidate ideas than on rejecting old ones. The key thing is to innovate until you hit a winner. Accordingly, decisions can be kept open as long as one tries to come up with additional candidates. At some point, of course, a candidate must be committed to. The process then moves to an iterative phase where incremental improvement is made. As has been noted, the base form arising from this approach is often a composite made from many failed attempts or parts of failed attempts. Only occasionally is it something that is completely new, based on parts and principles unlike anything that has come before.

### Would Either of These Highly General Approaches Fit the Time Shape of Ideas We Found Unfolding in Dance Creation?

The quick answer is “not really.” To properly distinguish the two would require more careful coding of modifications, since iterative design is described as incremental improvement on a seminal core. Unfortunately, “modification” was not a coding term our dancers used much, even when others might think it appropriate. As mentioned, and also noted in open-ended comments by the dancers, coding idea generation, and especially modification is difficult. When dancers move to create dance phrases and dance ideas, they explore dynamic postures, speed, shape, form, feeling, and movement across the floor and other aspects of dance. Phrase exploration includes all these things and more, since conscious goals, intentions, emotions, and constraints are present. The result is that unless dancers are practicing an already consolidated phrase their activities display change from episode to episode. Modification is hard to identify because it might be treated as consolidation or a simple reshaping of an existing idea. The open-ended comments from the dancers align with this view.

Looking at the data, a hint of an answer can be gleaned by reflecting on the rarity of rejecting an idea outside of the interval when it was first introduced. Of a total of 109 ideas generated, 75 were retained and 34 were rejected. Only two dancers on one occasion each rejected an earlier idea. About 94% of all rejections took place in the same interval they were created in. So, broadly speaking, ideas fail fast if they are going to fail at all.

The complication is that, in our dance study, only 31% of all ideas were rejected. More than twice as many ideas were kept. That is a high percentage and a great many ideas to retain. Somehow, the dancers incorporated numerous ideas into their final performance. So, a better characterization of our data is that, in dance, ideas fail fast but not often.

If the fail often model does not fit, can we see dance as closer to iterative design? Again, the data do not seem to support it. According to the dancers, their final product is not typically a good idea iteratively improved. It is made up of parts. As a 60 or 90 s product, it contains many different phrases or parts of phrases. It also had to be spatially, temporally, and compositionally edited – a process often called structuring (e.g., Gavish and Stevens, 2020). The final 60–90 s performance then is not best seen as modifications of one big idea. It is made up of multiple ideas.

The open-ended comments from the dancers reveal that often their perception of the “show” of material was that it was an extended phrase. The quantitative and qualitative accounts can be reconciled by considering the interaction with other dancers during improvisation (e.g., Torrents et al., 2011). Phrases are taught to others and then further manipulated. To teach or to show another, an extended chunked sequence may require segmentation by the dancer who is observing with the intention of learning. To the dancer generating a new material on their own, the “show” appears to them to be a single extended phrase, whereas in teaching and observing, sub-phrases become apparent.

Sometimes germ identification is hard because a kernel idea’s form, spatially, temporally, and semantically, need not resemble its mature development. Consider how the entries in a writer’s notebook re-emerge in poems or stories later. The initial idea of a plot, a scene, a paragraph, a sentence, or even a phrase may vary in most surface respects. There are times when the function of a diary entry – an idea germ – is to trigger a line of thought, not to give the creator a form to edit. This is especially true when an idea germ acts like an algorithm or method. As parameters vary, the form of output may change non-linearly. There need be no *evident* set of additions, deletions, extensions, simple substitutions – the typical forms of editing – that serve as the trajectory from germ to final form. Consider 7 and 2,401, and the similarity is 7^4^ = 2,401. The same non-linear change can occur when the germ is about something abstract – an idea about a type of movement, for example – that can be concretized in countless ways, sometimes in arms, sometimes in legs or torso.

### Limitations of the Study and Future Directions

Conducted with the dancers in the ADT studio, the study combined ecological validity with experimental control. Such a blend has both strengths and limitations. Strengths include the natural setting and the co-design of the research and coding with the Artistic Director, Associate Artistic Director, and the dancers. Having the dancers code and annotate their own material from video minimized reliance on memory and eliminated interpretation, misinterpretation, or judgments by another of quality or creativity.

Limitations include the relatively small sample size and the granularity of material that prevented identification of T-patterns (e.g., Castañer et al., 2009). The experiment was conducted with a particular dance company and the method could be applied to other ensembles and choreographers. Constraints include the three time intervals – the dancers commented that the intervals were adequate for stimulating ideas but less so for idea development. The dancers working alone was a feature of experimental control, but it was also noted as a constraint on idea development and possibly phrase segmentation.

In future studies, a further phase could be included to investigate whether a choreographer’s goals might vary, such as receiving more candidate ideas of good quality from dancers or fewer candidates of higher quality. If the choreographer requires many ideas, then the dancers must produce a great deal of material. Another possibility is that the choreographer prefers the generation of as many ideas as the dancers think are as good as they are able to create, and the choreographer then picks and chooses among these. On the latter view, the choreographer can never have enough ideas and the process is more like fail fast fail often or like a hierarchical system, where dancers are the generators and the choreographer is the pruner. In a future study, a focus on whether the choreographer incorporates many ideas into one idea may further illuminate the process although such a process is still not quite incrementalism. To examine generalization, the coding scheme and method could be introduced to other companies and choreographic approaches, and explorations by dancers and by choreographers also compared.

## Conclusion

Our goal in this study was to learn something about the temporal structure of creativity in creative dance. Neither the iterative view nor the fail fast fail often view tells us much about the actual time course. *Prima facie* one would assume that if the best designs are the outcome of iteration then there must have been a creative idea initially that was chosen early enough to permit iteration. However, this need not be true: the decision, as to when an idea crosses the threshold of acceptability and is deemed worth iterating on, may force subjects to reject many ideas. Moreover, the most creative part of an idea may come in the iteration phase when a small change opens up possibilities that were undreamt of initially.

We found that dancers do generate more good ideas in the early phase of a session and that though they prune these vigorously, approximately two-thirds of those original ideas find their way into the final performance of the day. Idea generation is significantly slower in subsequent periods and pruning is less vigorous, resulting in a higher percentage of later ideas being kept, though considerably fewer in absolute number.

It might seem that a higher rejection rate of ideas early in the process supports the popular view that it is desirable for creatives (and start-up businesses) to be agile, to take risks, to fail, and then pivot to new ideas. But, the high retention rate of ideas in the dance activity that we observed does not support this view.

The popular alternative view that creativity in design and other fields is best characterized as finding a good idea and then incrementally improving it also did not fit our data. Again, too many ideas were kept to justify seeing the final product as just a revision of one good idea. The result in dance is more compound, with more parts having more interaction.

Further studies are needed to introduce additional distinctions that may yield better insight into the interaction between time and idea generation, modification, and rejection.

## Data Availability Statement

The datasets generated for this study are available on request to the corresponding author.

## Ethics Statement

The studies involving human participants were reviewed and approved by Western Sydney University Human Research Ethics Committee. The participants provided their written informed consent to participate in this study and for the publication of images or data included in this article.

## Author Contributions

DK designed the research question and experiment, guided the data analyses, and drafted the manuscript. CS conducted the experiment, coordinated data collection and analysis, and contributed to the literature review and manuscript. DP organized the data and conducted the statistical analyses. All authors contributed to the article and approved the submitted version.

### Conflict of Interest

The authors declare that the research was conducted in the absence of any commercial or financial relationships that could be construed as a potential conflict of interest.
